# Trends in the Epidemiology and Outcomes of Pneumocystis Pneumonia among Human Immunodeficiency Virus (HIV) Hospitalizations

**DOI:** 10.3390/ijerph19052768

**Published:** 2022-02-27

**Authors:** Kalaimani Elango, Mayuri Mudgal, Swetha Murthi, Prashanth Reddy Yella, Savan Nagrecha, Vedhapriya Srinivasan, Vijaykumar Sekar, Maria Koshy, Sathishkumar Ramalingam, Kulothungan Gunasekaran

**Affiliations:** 1 Division of Cardiology, University of Nevada, 4505 S Maryland Pkwy, Las Vegas, NV 89154, USA; kalaimani.elango@gmail.com; 2 Department of Geriatric Medicine, Montefiore Medical Center, Wakefield Campus, 600 E 233rd Street Bronx, New York, NY 10466, USA; mayurimudgal@gmail.com; 3 Department of Endocrinology, Yuma Regional Medical Center, 2400 S Avenue A, Yuma, AZ 85364, USA; drswethamurthi@gmail.com; 4 Department of Internal Medicine, Yuma Regional Medical Center, 2400 S Avenue A, Yuma, AZ 85364, USA; prashanthreddy2179@gmail.com; 5 Department of Pharmacy, Yuma Regional Medical Center, 2400 S Avenue A, Yuma, AZ 85364, USA; savanrx@gmail.com; 6 Department of Internal Medicine, Suny Downstate Medical Center, New York, NY 11203, USA; vedhavimal612@gmail.com; 7 Department of Endocrinology, Lehigh Valley Health Center, 1243 S Cedar Crest Blvd, Allentown, PA 18103, USA; medvijay1983@gmail.com; 8 Department of Internal Medicine, Bridgeport Hospital, 267 Grant Street, Bridgeport, CT 06610, USA; shrutikoshy@gmail.com; 9 Department of Internal Medicine, Lovelace Medical Center, 601 Dr. Martin Luther King Jr. Avenue NE, Albuquerque, NM 87102, USA; sathishmed@gmail.com; 10 Department of Pulmonary Diseases and Critical Care, Yuma Regional Medical Center, 2400 S Avenue A, Yuma, AZ 85364, USA

**Keywords:** *Pneumocystis* pneumonia, PCP, human immunodeficiency virus, HIV, epidemiology, *Pneumocystis jirovecii*, opportunistic infection, antiretroviral therapy, pneumonia

## Abstract

Introduction: *Pneumocystis* Pneumonia (PCP) is a common opportunistic infection among people living with the human immunodeficiency virus (HIV). This study’s objective was to assess temporal trends in PCP epidemiology among hospitalized patients with HIV/AIDS in the US and to compare data for hospitalizations with HIV with PCP to those without PCP. Methods: The national inpatient sample (NIS) data were analyzed from 2002–2014. The discharge coding identified hospitalized patients with HIV or AIDS and with or without PCP. Results: We identified 3,011,725 hospitalizations with HIV/AIDS during the study period; PCP was present in 5% of the patients with a diagnosis of HIV. The rates of PCP progressively declined from 6.7% in 2002 to 3.5 % in 2014 (*p* < 0.001). Overall mortality in patients with HIV was 3.3% and was significantly higher in those with PCP than without PCP (9.9% vs. 2.9%; *p* < 0.001). After adjusting for demographics and other comorbidities, PCP had higher odds of hospital mortality 3.082 (OR 3.082; 95% CI, 3.007 to 3.159; *p* < 0.001). Conclusion: From 2002 to 2014, the rate of PCP in HIV patients has decreased significantly in the United States but is associated with substantially higher mortality.

## 1. Introduction

Human immunodeficiency virus (HIV)/acquired immune-deficiency syndrome (AIDS) continues to be a significant health burden with around 36 million people worldwide [1,2]. Of this, approximately 1.2 million HIV-positive people reside in USA [3]. Pneumocystis pneumonia has long been known to be a significant health concern in this sub-population.

A fungus, *Pneumocystis jirovecii,* causes Pneumocystis pneumonia *(PCP)*. With changes in the taxonomy, *Pneumocystis carinii* now denotes *Pneumocystis* that infects rats, and *P. jirovecii* denotes the species that infects humans.

Before early 1980s, *Pneumocystis carini* pneumonia (PCP) was a rare but fatal infection that occurred in immune-compromised patients with hematopoietic cancers (predominantly, acute lymphocytic leukemia), those with protein energy malnutrition, or in patients receiving corticosteroid therapy. In 1981, a report of five cases of *Pneumocystis carinii* pneumonia (PCP) among previously healthy homosexual young men in Los Angeles was noted [4]. This first identified the initial signs of the acquired immunodeficiency syndrome (AIDS) epidemic in HIV patients.

Approximately 90% of PCP cases occurred in patients with CD4 T lymphocyte (CD4) cell counts <200 cells/mm^3^. Other risk factors for PCP in the pre-ART (Anti-retroviral therapy) era were CD4 cell percentage <14%, previous episodes of PCP, oral thrush, recurrent bacterial pneumonia, unintentional weight loss, and higher plasma HIV RNA levels [5,6].

The clinical triad of PCP includes subacute onset of progressive dyspnea, fever, and a non-productive cough. There is chest discomfort that worsens within days to weeks [7].

The earlier diagnosis of HIV, antiretroviral therapy, and effective prophylaxis have all contributed to a 75% decline in PCP cases [8]. However, the rates of hospitalization among HIV-positive persons continue to remain higher than that of the general population [9,10].

In one study, HIV-infected patients with PCP had a significantly greater number of organisms and fewer neutrophils in bronchoalveolar lavage (BAL) fluid, and less severe oxygenation impairment compared to other immunocompromised patients with PCP. This proposed that the severity of PCP could be determined by the inflammatory response rather than by the load of the organism itself [11]. Other studies have compared PCP features and outcomes in HIV versus non-HIV patients suggesting increased requirement of intensive care or ventilation, and the rate of in-hospital deaths [12,13]. We aim to determine the impact of PCP on patients with HIV admitted to the hospital and assess temporal trends in the patients with PCP versus those without PCP.

## 2. Materials and Methods

### 2.1. Data Source and Study Design

This study was a retrospective cross-sectional study of all hospital admissions with a primary discharge diagnosis of human immunodeficiency virus (HIV) between 2002 and 2014. We examined the trends in the epidemiology and outcomes of Pneumocystis pneumonia (PCP) among HIV hospitalizations using discharge data from the National Inpatient Sample (NIS), the Healthcare Cost and Utilization Project (HCUP), and the Agency for Healthcare Research and Quality [14]. As the largest publicly available all-payer inpatient healthcare database in the USA, the NIS provides national estimates of more than 7 million discharges per annum. NIS is a self-weighted, stratified, systematic, random sample of 20% discharges from all non-federal US community hospitals (prior to 2012, it was a 20% sample of hospitals from which all discharges were retained). To account for this redesign, the trend weight (TRENDWT) provided by HCUP is used in place of original discharge weight (DISCWT) to create national estimates for years prior to 2012 [15]. The NIS sample is stratified on hospital characteristics. This form of clustering tends to induce dependence among discharges within hospitals; hence, variance analysis of subsets in line with NIS methods was performed [16]. The diagnoses and procedures were encoded with the International Classification of Diseases, Ninth Revision, Clinical Modification (ICD-9-CM). Since this study included deidentified data, per the data use agreement with the Agency for Healthcare Research and Quality, the institutional review board requirement was waived. Hospital charges were included as “Total charges” incurred during the admission as provided by the NIS database.

### 2.2. Study Groups and Outcomes

We used *ICD-9-CM* codes to identify all hospitalized adults (aged ≥ 18 years), who had a primary diagnosis of HIV or AIDS (*ICD-9-CM* diagnosis codes 042×, 043×, 044×, 079.53, 795.71, V08, V65, 44,042.9) between January 2002 and December 2014. Those with missing data for gender, mortality, and length of stay were excluded. Dummy cases with hospital identifiers were added to make sure all the hospitals in the US irrespective of HIV diagnosis were added to the analysis to account for the complex sampling design of the NIS database given the clustering effect.

Among HIV/AIDS admissions, those with PCP were extracted using *ICD-9 CM* diagnosis code 136.3 from the secondary diagnoses. NIS provides 29 comorbidities (also known as Elixhauser comorbidity measures) based on *ICD-9 CM* diagnoses and the diagnosis-related group in effect on the date of discharge. These comorbidities are not directly related to the principal diagnosis or the main reason for admission and are likely to have originated before the hospital stay [17].

The primary outcome of interest was the trends in the prevalence and outcomes such as hospital length of stay, and in-hospital mortality among 2 subgroups of HIV, such as HIV with PCP, and HIV with no PCP.

### 2.3. Statistical Analyses

We adhered to the methodological standards described by Khera et al. [18]. All data analyses were performed using IBM SPSS Statistics for Windows, version 24.0 (IBM, Armonk, NY, USA). The study analysis was performed using the complex sample analysis method accounting for the clustering effect of the sample design. Weight was applied to obtain national estimates. Continuous values are reported as mean ± standard error of the mean and compared using analysis of variance (ANOVA). Categorical variables were reported as a number and/or percentage and compared using the chi-squared test. Multivariable logistic regression analyses were performed while accounting for the sampling technique and adjusting for various demographic variables, clinical variables, and hospital characteristics.

## 3. Results

### 3.1. PCP Incidence, Demographic Characteristics and Prevelance

**Figure 1:**
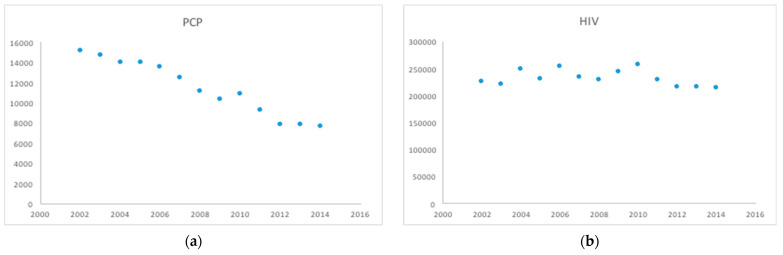
Trends of hospitalization Pneumocystis pneumonia in HIV (**a**) and HIV admissions in general (**b**) 2002–2014.

From January 2002 to December 2014, there were an estimated 3,011,725 hospitalizations with HIV/AIDS. Out of these, 148,624 patients (5%) had PCP and 2,863,099 patients (95%) did not have PCP. The mean age for HIV patients with PCP was 42 ± 10 years and without PCP was 45 ± 11 years. Among HIV hospitalizations, males had a higher prevalence of PCP compared to females (70% vs. 30%, *p* < 0.0001). The majority of HIV hospitalizations with and without PCP were Blacks (52.7% and 53.3% respectively) followed by Caucasians (27.9% and 24.8% respectively) followed by Hispanics (13.8% and 12.3% respectively). The mean LOS in days was lower in HIV hospitalizations without PCP (6.33 vs. 10.59, *p* < 0.0001), suggesting a longer hospital stay in HIV patients with PCP. The majority of the HIV hospitalizations both with PCP and without PCP were in an urban setting (96.5% vs. 3.5%, *p* < 0.0001) (Table 1). Over a period of 13 years, we noted a progressive decline in rates of PCP among HIV hospitalizations from 6.7% in 2002 to 3.5% in 2014 (*p* < 0.0001) (Table 2 and Figure 1).

Multivariate logistic regression analyses were performed. In the multivariate analysis, prevalence, length of stay and in-hospital mortality were analyzed. All models were adjusted for age, gender, race, comorbidities, residential region, hospital size, location/teaching status of the hospital, and median household income (Table 1).

### 3.2. Prevalence

Our study results show that the prevalence of PCP in patients with HIV hospitalizations declined over the period of 13 years (Table 2 and Figure 1). HIV patients with chronic pulmonary disease, deficiency anemias, fluid and electrolyte disorders, and weight loss had a higher prevalence of PCP compared to HIV patients with other comorbidities (Table 1).

### 3.3. Length of Stay Outcome

The mean length of stay for all HIV hospitalizations was 6.54 ± 8.93 days. When length of stay was compared among the groups, HIV patients with PCP had a longer hospital stay than HIV patients without PCP (10.59 vs. 6.33, *p* < 0.0001). This was evident in the total charges incurred during the hospital stay among the patient population. Those patients without PCP had a total cost of about $36,860.45. This was much lower than the total charges incurred for the HIV patients with PCP which was about $65,090.83 (*p* < 0.0001) (Table 1).

### 3.4. In-Hospital Mortality Outcome

Overall, in-hospital mortality in patients with HIV was 3.3% and was significantly higher in HIV patients with PCP than without PCP (9.9% vs. 2.9%; *p* < 0.001) (Table 1). After adjusting for demographics and other comorbidities, PCP was associated with higher odds of hospital mortality 3.082 (OR 3.082, [95% confidence interval, CI: 3.007 to 3.159]; *p* < 0.001) among HIV hospitalizations. Among the other comorbidities, congestive heart failure, coagulopathy, liver disease, lymphoma, fluid, and electrolyte disorders, metastatic cancer, pulmonary circulation disorders, renal failure, solid tumors, and weight loss were associated with higher odds of in-hospital mortality among HIV patients (Table 3). Urban teaching and large-sized hospitals were associated with higher odds of mortality when compared to rural (OR 1.081 [95% CI: 1.044–1.120]; *p* < 0.0001) and small-sized hospitals (OR 1.038 [95% CI: 1.018–1.058]; *p* < 0.0001), respectively (Table 3). Interestingly, the percentage of in-hospital mortality in HIV patients with PCP declined every year from 2002 to 2014 (11.5 % to 8.4%) (Table 4).

## 4. Discussion

*Pneumocystis* pneumonia (PCP) is an AIDS-defining illness [19]. In the Antiretroviral Therapy Cohort Collaboration, comprised of 15 North American and European cohorts established in 2000, PCP was the second most frequent AIDS-defining diagnosis (first being esophageal candidiasis) [20]. Various studies have noted approximately 23–31% PCP cases occurring in patients who were newly diagnosed with HIV at the time of PCP presentation [21,22,23]. Other cases occurred with non-adherence to anti-retroviral therapy (ART) or sub-optimal immune recovery [24]. Another study from Spain noted an increase in the proportion of patients with PCP, before being diagnosed with HIV, from 48% in 2000 to 67% in 2013 [25]. PCP, therefore, continues to remain an important health concern in patients with HIV.

Our study noted a decline in PCP prevalence amongst HIV patients from 6.7% in 2002 to 3.5% in 2014 with a cumulative prevalence of 5%. A similar trend was demonstrated by the Centers for Disease Control and Prevention, AIDS Surveillance Summaries, 1989–1992, where the prevalence of PCP in HIV patients was noted at 53% in 1989 with a subsequent decline to 42% by 1992 and 3.4% every year from 1992 [26]. Sub-Saharan studies from 1992–1994 were notable for PCP prevalence of 5–9%, which was relatively lower compared to developed countries at that time [27,28,29]. This was noted to be likely secondary to neglected/missed data, as proven by a systematic review and meta-analysis of 48 sub-Saharan studies. This meta-analysis also noted a decrease in the prevalence of PCP in HIV-infected patients in sub-Saharan Africa between 1995 and 2015 from 28% to 9% after 2005 [30].

Of note, prophylaxis against pneumocystis with trimethoprim–sulfamethoxazole was introduced in 1989 [31]. Various trials have noted that this once-a-day pill has been highly effective in the primary prevention as well as treatment for PCP [32,33]. The combined antiretroviral therapy was approved in 1991 when additional nucleoside reverse transcriptase inhibitors (NRTI) were approved by the FDA for HIV infection (NRTI, Zidovudine was approved in 1987) which was followed by approval of the non-nucleoside class of antiretrovirals in 1995, allowing for the possible use of combination antiretrovirals called highly active anti-retroviral therapy (HAART) to treat HIV disease. The Global Burden of Disease [GBD] 2017 HIV collaborators noted that the access to ART from 2.98 million people in 2006 to 21.8 million in 2017 was accompanied by a 51% reduction in HIV mortality, from 1.95 million in 2006 to 0.95 million in 2017. Interestingly, in 2006, a single-tablet regimen for ART was introduced. This was followed by multiple single-tablet regimens. A 2014 meta-analysis emphasized that better adherence and virological suppression were achieved with this regimen [34].

Similarly, an increased availability and improved adherence as once a day medication of trimethoprim–sulfamethoxazole for PCP prophylaxis and treatment along with single-tablet regimen for ART explains that probably fewer HIV patients need to be hospitalized for management of PCP, thus decreasing its prevalence in our study population.

HIV with/without PCP was predominantly common in males with lower median household income and in African American, white, and Hispanic populations in our study. This has also been noted in literature before. This is likely secondary to higher rates of male-to-male sexual contact as well as infrequent HIV testing and late HIV diagnosis among these populations suffering from healthcare disparities [35]. This also corresponds to an increased prevalence of HIV and PCP infections in the southern and northeast regions of USA as noted in our study.

As per Centers for Disease Control and Prevention (CDC), the rates of HIV incidence are highest among ages 25–44 [3]. In one study, approximately 9.4% of newly diagnosed HIV patients, were more than 55 years of age. Diagnosis delay (time from infection to diagnosis) was longer among persons who were older at diagnosis than among those who were younger (median = 4.5 years among persons aged ≥55 years compared with 2.4 years among persons aged 13–24 years) (*p* < 0.01). This meant that older population are more likely to have a higher stage of HIV at the time of diagnosis [36]. Moreover, heterosexual men are more likely than women and men having sex with men to have missed the opportunity for getting tested for HIV therefore having a longer diagnostic delay [37]. Similarly, non-white racial/ethnic groups had higher proportion of infections attributable to heterosexual contact among these groups compared with whites [38].

These factors denote that HIV patients with diagnostic delays have a higher immune function damage at the time of diagnosis which increases their morbidity and mortality. Earlier diagnosis along with prompt linkage to health care and initiation of antiretroviral treatment enhances improvement of immune function and viral suppression thereby, reducing risk for sexual transmission of HIV [39].

The in-hospital mortality rate for patients with HIV and PCP has been in the range of 4–32% [40,41]. In our multi-variate logistic regression analysis adjusting for patient demographics and co-morbidities, there is an increased mortality in HIV patients with PCP compared to HIV without PCP (9.9% vs. 2.9%, OR 3.08). Mortality risk factors for HIV patients with PCP have previously been identified and include increasing age, subsequent episodes of PCP, low hemoglobin level, low basal PaO2 at hospital admission, pulmonary Kaposi sarcoma, and pre-existing co-morbidities [3,40]. A study from Taiwan noted three predictors associated with mortality-systolic blood pressure ≤110 mmHg [adjusted odds ratio (AOR) 3.88; 95% confidence interval (CI) 1.17–12.83; *p* = 0.03], PaO2at room air ≤ 60 mmHg (AOR 4.97; 95% CI 1.34–18.23; *p* = 0.01), and lymphocytes ≤ 10% (AOR 8.19; 95% CI 1.48–45.36; *p* = 0.02) [42]. Another study from China noted initial lactate dehydrogenase levels (level more than 495 U/L with sensitivity and specificity of 70%) to be an independent predictor for mortality in this sub-population of patients [43].

Weight loss, congestive heart failure, coagulopathy, metastatic cancer, liver disease, and renal failure were also noted to be associated with increased mortality in our HIV population. Advanced AIDS patients experience significant weight loss, have higher coagulopathy, an increased risk of AIDS related/non-AIDS related cancers, and increased chances of concomitant Hepatitis C infection affecting the liver as well sepsis related renal failure. This is also suggested in prior studies, which noted increased non-AIDS deaths (from non-AIDS infections, cardiovascular, and non-AIDS malignancy) during the HAART era [44,45]. It is believed that these are due to complications of aging HIV population, adverse effects of HAART as well as from HIV infection induced unregulated inflammatory response while on HAART accelerating these co-morbidities [46,47].

HIV patients with PCP also have a longer length of stay and higher hospital costs. This is likely because these patients were sicker (possibly due to diffuse alveolar damage due to the host’s own inflammatory response causing significant lung injury and defective gas exchange) compared to HIV alone and therefore leading to increased mortality in this sub-population.

The weekend effect, which has been observed with various other medical conditions, was present in this study as well [48,49,50]. There was an 11% increase in the risk of weekend mortality compared to weekday mortality in HIV patients with PCP.

## 5. Limitations

Our study has several limitations, some of which are inherent to the analysis of a large administrative database. Since NIS data is based on the discharge diagnosis for identifying patients, and not on individual patients, errors in coding could have led to missing data. The data on the medications, laboratory values are not provided by the database. [50,51] Whether the HIV patients getting hospitalized had already initiated an outpatient prophylaxis/treatment, or had a different inpatient encounter for PCP, could not be ascertained during this admission [50,51,52]. It is also possible that this subset of hospitalized HIV patients with PCP had failed outpatient empiric antibiotic treatment for other etiologies of pneumonia. Some patients might have had a mild PCP infection not requiring hospitalization. Despite these limitations, this study highlights the prevalence of PCP in HIV patients requiring inpatient treatment and addresses the impact of PCP burden in this subset of hospitalized population.

## 6. Conclusions

In summary, PCP is a life-threatening condition in HIV patients with significantly higher mortality, however, the prevalence of PCP and the mortality from PCP in HIV patients have decreased significantly in the HAART era. Our study re-emphasizes the need for early HIV case detection and continued care to prevent morbidity and mortality from AIDS-defining illnesses, specifically PCP.

## Figures and Tables

**Table 1:** Demographic characteristics of HIV hospitalizations with and without Pneumocystis pneumonia (PCP).

Clinical Characteristics	All HIV (*n* = 3,011,724)	HIV without PCP (*n* = 2,863,099)	HIV with PCP (*n* = 148,624)	*p*-Value
Age	45.18 ± 11.109	45.35 ± 11.133	42.08 ± 10.141	<0.0001
Length of stay	6.54 ± 8.938	6.33 ± 8.742	10.59 ± 11.378	<0.0001
Total charges	38,243.79 ± 66,337	36,860.45 ± 63,561	65,090.83 ± 103,153	<0.0001
Sex				
Male	1,981,195 (65.8%)	1,876,654 (65.5%)	104,542 (70.3%)	<0.0001
Female	1,030,528 (34.2%)	986,446 (34.5%)	44,083 (29.7%)	<0.0001
Race (uniform)				
White	745,951 (24.8%)	711,292 (27.9%)	34,659 (26.8%)	<0.0001
Black	1,426,627 (47.4%)	1,358,620 (53.3%)	68,006 (52.7%)	<0.0001
Hispanic	370,092 (12.3%)	350,334 (13.8%)	19,758 (15.3%)	<0.0001
Asian or Pacific Islander	15,160 (0.5%)	13,781 (0.5%)	1379 (1.1%)	<0.0001
Native American	9370 (0.3%)	8853 (0.3%)	517 (0.4%)	<0.0001
Others	108,755 (3.6%)	103,914 (4.1%)	4841 (3.7%)	<0.0001
Region of Hospital				
Northeast	991,494 (32.9%)	957,493 (33.4%)	34,001 (22.9%)	<0.0001
Midwest or North Central	367,812 (12.2%)	352,235 (12.3%)	15,577 (10.5%)	<0.0001
South	1344,453 (44.6%)	1,265,753 (44.2%)	78,701 (53%)	<0.0001
West	307,964 (10.2%)	287,618 (10%)	20,346 (13.7%)	<0.0001
Died	98,361 (3.3%)	83,666 (2.9%)	14,695 (9.9%)	<0.0001
Admission day is a weekend	634,783 (21.1%)	601,454 (21%)	33,328 (22.4%)	<0.0001
Disposition of patient (uniform)				
Routine	2,108,095 (70%)	2,008,111 (70.1%)	99,984 (67.3%)	<0.0001
Short-term hospital	53,929 (1.8%)	51,218 (1.8%)	2712 (1.8%)	<0.0001
Skilled Nursing Facility (SNF)	332,526 (11%)	318,668 (11.1%)	13,858 (9.3%)	<0.0001
Intermediate Care Facility (ICF)	252,057 (8.4%)	241,519 (8.4%)	10,538 (7.1%)	<0.0001
Another type of facility	165,155 (5.5%)	158,414 (5.5%)	6741 (4.5%)	<0.0001
Home Health Care (HHC)	98,361 (3.3%)	83,666 (2.9%)	14,695 (9.9%)	<0.0001
Against medical advice (AMA)	1600 (0.1%)	1503 (0.1%)	97 (0.1%)	<0.0001
Elective admission	352,729 (11.7%)	345,138 (12.1%)	7591 (5.1%)	<0.0001
Primary expected payer (uniform)				
Medicare	878,453 (29.2%)	853,848 (29.9%)	24,604 (16.7%)	<0.0001
Medicaid	1,222,318 (40.6%)	1,164,708 (40.8%)	57,610 (39%)	<0.0001
Private insurance	484,582 (16.1%)	451,652 (15.8%)	32,930 (22.3%)	<0.0001
Self-pay	267,607 (8.9%)	245,845 (8.6%)	21,761 (14.7%)	<0.0001
No charge	40,276 (1.3%)	37,399 (1.3%)	2877 (1.9%)	<0.0001
Other	109,624 (3.6%)	101,666 (3.6%)	7958 (5.4%)	<0.0001
Median household income quartile for patient’s ZIP Code				
0–25th percentile	1,298,141 (43.1%)	1,233,011 (49.5%)	65,130 (48.1%)	<0.0001
26th to 50th percentile (median)	603,834 (20%)	572,324 (23%)	31,510 (23.3%)	<0.0001
51st to 75th percentile	446,650 (14.8%)	422,667 (17%)	23,983 (17.7%)	<0.0001
76th to 100th percentile	280,062 (9.3%)	265,201 (10.6%)	14,861 (11%)	<0.0001
Bed size of hospital				
Small	273,420 (9.1%)	261,546 (9.2%)	11,873 (8%)	<0.0001
Medium	748,044 (24.8%)	710,475 (24.9%)	37,569 (25.4%)	<0.0001
Large	1,979,628 (65.7%)	1,880,911 (65.9%)	98,718 (66.6%)	<0.0001
Control/ownership of hospital				
Government or private (collapsed category)	1,825,278 (60.6%)	1,732,348 (77.6%)	92,930 (74.4%)	<0.0001
Government, nonfederal (public)	113,581 (3.8%)	106,306 (4.8%)	7275 (5.8%)	<0.0001
Private, not-for-profit (voluntary)	234,390 (7.8%)	219,797 (9.8%)	14,593 (11.7%)	<0.0001
Private, investor-owned (proprietary)	172,701 (5.7%)	163,042 (7.3%)	9658 (7.7%)	<0.0001
Private (collapsed category)	11,387 (0.4%)	10,929 (0.5%)	458 (0.4%)	<0.0001
Location/teaching status of hospital				
Rural	103,534 (3.4%)	98,666 (3.5%)	4869 (3.3%)	<0.0001
Urban nonteaching	803,918 (26.7%)	762,579 (26.7%)	41,338 (27.9%)	<0.0001
Urban teaching	2,093,640 (69.5%)	1,991,687 (69.8%)	101,953 (68.8%)	<0.0001
Alcohol abuse	278,873 (9.3%)	269,281 (9.5%)	9592 (6.5%)	<0.0001
Deficiency anemias	670,353 (22.3%)	622,040 (21.9%)	48,314 (32.7%)	<0.0001
Rheumatoid arthritis/collagen vascular diseases	20,064 (0.7%)	19,553 (0.7%)	512 (0.3%)	<0.0001
Chronic blood loss anemia	37,557 (1.2%)	36,512 (1.3%)	1045 (0.7%)	<0.0001
Congestive heart failure	141,072 (4.7%)	134,102 (4.7%)	6970 (4.7%)	<0.0001
Chronic pulmonary disease	536,730 (17.8%)	508,861 (17.9%)	27,869 (18.9%)	<0.0001
Coagulopathy	226,624 (7.5%)	215,079 (7.6%)	11,544 (7.8%)	<0.0001
Depression	323,648 (10.7%)	311,747 (11%)	11,901 (8.1%)	<0.0001
Diabetes, uncomplicated	324,690 (10.8%)	315,834 (11.1%)	8855 (6%)	<0.0001
Diabetes with chronic complications	65,236 (2.2%)	63,743 (2.2%)	1493 (1%)	<0.0001
Drug abuse	640,836 (21.3%)	615,264 (21.6%)	25,572 (17.3%)	<0.0001
Hypertension	935,590 (31.1%)	909,666 (32%)	25,924 (17.5%)	<0.0001
Hypothyroidism	81,825 (2.7%)	79,423 (2.8%)	2403 (1.6%)	<0.0001
Liver disease	351,442 (11.7%)	339,904 (11.9%)	11,538 (7.8%)	<0.0001
Lymphoma	74,646 (2.5%)	72,198 (2.5%)	2448 (1.7%)	<0.0001
Fluid and electrolyte disorders	815,607 (27.1%)	752,205 (26.4%)	63,402 (42.9%)	<0.0001
Metastatic cancer	31,004 (1%)	30,498 (1.1%)	506 (0.3%)	<0.0001
Other neurological disorders	237,746 (7.9%)	230,051 (8.1%)	7695 (5.2%)	<0.0001
Obesity	101,271 (3.4%)	98,513 (3.5%)	2758 (1.9%)	<0.0001
Paralysis	56,265 (1.9%)	55,135 (1.9%)	1130 (0.8%)	<0.0001
Peripheral vascular disorders	47,390 (1.6%)	46,691 (1.6%)	699 (0.5%)	<0.0001
Psychoses	248,541 (8.3%)	240,240 (8.4%)	8301 (5.6%)	<0.0001
Pulmonary circulation disorders	40,013 (1.3%)	37,177 (1.3%)	2835 (1.9%)	<0.0001
Renal failure	361,463 (12%)	351,618 (12.4%)	9845 (6.7%)	<0.0001
Solid tumor without metastasis	44,766 (1.5%)	43,738 (1.5%)	1028 (0.7%)	<0.0001
Peptic ulcer disease excluding bleeding	2417 (0.1%)	2301 (0.1%)	116 (0.1%)	<0.0001
Valvular disease	48,602 (1.6%)	46,589 (1.6%)	2013 (1.4%)	<0.0001
Weight loss	218,633 (7.3%)	194,137 (6.8%)	24,496 (16.6%)	<0.0001

**Table 2:** Trends of hospitalization for HIV and Pneumocystis pneumonia in HIV 2002–2014.

Calendar Year	Total Number of HIV Hospitalizations	Total Number of HIV Hospitalizations with PCP
2002	225,202	15,144
2003	220,627	14,682
2004	248,090	14,006
2005	229,448	13,982
2006	253,354	13,535
2007	233,297	12,489
2008	228,136	11,138
2009	243,649	10,342
2010	257,093	10,820
2011	229,071	9240
2012	216,110	7800
2013	214,685	7780
2014	212,960	7665

**Table 3:** Multivariate logistic regression analysis showing the adjusted odds ratios predicting the in-hospital mortality for HIV hospitalizations.

HIV Patients with Comorbidities	Odds Ratio for Mortality	95% Confidence Interval		*p*-Value
Pneumocystis pneumonia	3.082	3.007	3.159	<0.0001
Alcohol abuse	0.799	0.773	0.827	<0.0001
Deficiency anemias	0.715	0.701	0.73	<0.0001
Rheumatoid arthritis/collagen vascular diseases	0.86	0.759	0.975	<0.0001
Chronic blood loss anemia	1.054	0.984	1.129	<0.0001
Congestive heart failure	1.834	1.778	1.892	<0.0001
Chronic pulmonary disease	0.789	0.77	0.809	<0.0001
Coagulopathy	3.54	3.467	3.615	<0.0001
Depression	0.551	0.531	0.572	<0.0001
Diabetes, uncomplicated	0.897	0.871	0.924	<0.0001
Diabetes with chronic complications	0.786	0.74	0.835	<0.0001
Drug abuse	0.679	0.663	0.696	<0.0001
Hypertension	0.675	0.66	0.69	<0.0001
Hypothyroidism	0.97	0.919	1.023	<0.0001
Liver disease	1.388	1.356	1.421	<0.0001
Lymphoma	2.574	2.485	2.667	<0.0001
Fluid and electrolyte disorders	2.904	2.854	2.955	<0.0001
Metastatic cancer	3.787	3.601	3.983	<0.0001
Other neurological disorders	1.845	1.799	1.893	<0.0001
Obesity	0.651	0.604	0.702	<0.0001
Paralysis	1.357	1.284	1.434	<0.0001
Peripheral vascular disorders	1.256	1.177	1.342	<0.0001
Psychoses	0.653	0.627	0.68	<0.0001
Pulmonary circulation disorders	1.811	1.711	1.916	<0.0001
Renal failure	1.811	1.766	1.856	<0.0001
Solid tumor without metastasis	1.657	1.567	1.752	<0.0001
Peptic ulcer disease excluding bleeding	0.828	0.648	1.058	<0.0001
Valvular disease	1.027	0.971	1.086	<0.0001
Weight loss	1.873	1.829	1.918	<0.0001

**Table 4:** Trends of mortality in HIV patients with PCP from 2002–2014.

Calendar Year	Mortality in PCP	Mortality in PCP (%)
2002	1736	11.5
2003	1572	10.7
2004	1590	11.4
2005	1394	10
2006	1393	10.3
2007	1126	9
2008	1153	10.4
2009	1010	9.8
2010	945	8.7
2011	685	7.4
2012	685	8.8
2013	760	9.8
2014	645	8.4

## Data Availability

The data presented in this study are available on request from the corresponding author.
